# Evaluation of a person-centred, nurse-led model of care delivering hepatitis C testing and treatment in priority settings: a mixed-methods evaluation of the Tasmanian Eliminate Hepatitis C Australia Outreach Project, 2020–2022

**DOI:** 10.1186/s12889-023-17066-9

**Published:** 2023-11-20

**Authors:** Joshua Dawe, Megan Hughes, Shannon Christensen, Louisa Walsh, Jacqueline A. Richmond, Alisa Pedrana, Anna L. Wilkinson, Louise Owen, Joseph S. Doyle, on behalf of the EC Australia Partnership, Margaret Hellard, Mark Stoove, Nick Scott, Jess Howell, Linda Selvey, Jessica Michaels, Sione Crawford, Carrie Fowlie, Shweta Singhal, Jane Davies, Geoff Manu, James Ward, Geoff Drenkhahn, Lisa Bastian, Greg Dore, Mellissa Bryant, Catherine Marshall, Andrew Llyod, Maria McMahon, Garry Sattell, Dawn Casey, David Shaw, Tom Rees, Alex Thompson

**Affiliations:** 1 Disease Elimination, Burnet Institute, Melbourne, Australia; 2 Sexual Health Service Tasmania, Hobart, Australia; 3 Centre for Health Communication and Participation, La Trobe University, Melbourne, Australia; 4 School of Public Health and Preventive Medicine, Monash University, Melbourne, Australia; 5 Melbourne School of Population and Global Health, University of Melbourne, Melbourne, Australia; 6 Department of Infectious Diseases, Alfred Health and Monash University, Melbourne, Australia

**Keywords:** Hepatitis C, Direct-acting antivirals, Nurse-led model of care, People who inject drugs, Australia

## Abstract

**Introduction:**

Australia has experienced sustained reductions in hepatitis C testing and treatment and may miss its 2030 elimination targets. Addressing gaps in community-based hepatitis C prescribing in priority settings that did not have, or did not prioritise, hepatitis C testing and treatment care pathways is critical.

**Methods:**

The Tasmanian Eliminate Hepatitis C Australia Outreach Project delivered a nurse-led outreach model of care servicing hepatitis C priority populations in the community through the Tasmanian Statewide Sexual Health Service, supported by the Eliminating Hepatitis C Australia partnership. Settings included alcohol and other drug services, needle and syringe programs and mental health services. The project provided clients with clinical care across the hepatitis C cascade of care, including testing, treatment, and post-treatment support and hepatitis C education for staff.

**Results:**

Between July 2020 and July 2022, a total of 43 sites were visited by one Clinical Nurse Consultant. There was a total of 695 interactions with clients across 219 days of service delivery by the Clinical Nurse Consultant. A total of 383 clients were tested for hepatitis C (antibody, RNA, or both). A total of 75 clients were diagnosed with hepatitis C RNA, of which 95% (71/75) commenced treatment, 83% (62/75) completed treatment and 52% (39/75) received a negative hepatitis C RNA test at least 12 weeks after treatment completion.

**Conclusions:**

Providing outreach hepatitis C services in community-based services was effective in engaging people living with and at-risk of hepatitis C, in education, testing, and care. Nurse-led, person-centred care was critical to the success of the project. Our evaluation underscores the importance of employing a partnership approach when delivering hepatitis C models of care in community settings, and incorporating workforce education and capacity-building activities when working with non-specialist healthcare professionals.

**Supplementary Information:**

The online version contains supplementary material available at 10.1186/s12889-023-17066-9.

## Introduction

Globally, 58 million people live with chronic hepatitis C, with the greatest burden of disease concentrated among people who inject drugs, particularly in middle and high income countries [1]. In Australia, injecting drug use is a primary risk factor for incident hepatitis C infections [2]. With high cure rates, short treatment durations and few side effects, the development of direct-acting antiviral (DAA) therapy represents a major advancement in the hepatitis C testing and treatment landscape, and an opportunity to eliminate hepatitis C as a public health threat by 2030 [3, 4]. The hepatitis C cascade of care represents the sequential steps through which people living with hepatitis C are diagnosed and treated, and includes hepatitis C antibody screening, hepatitis C RNA confirmation testing, DAA treatment initiation and completion, and sustained virologic response (SVR). Recent evidence suggests that gaps in the hepatitis C cascade of care have persisted in the DAA era, particularly among people who inject drugs [5]. Consequently, the availability of DAAs alone are not sufficient for achieving hepatitis C elimination, and further efforts to enhance the provision of hepatitis C care are required.

Australian citizens and permanent residents have access to Medicare, Australia’s tax-payer funded, universal healthcare system [6]. Medicare provides free or low-cost access to many medical services, including hospital, specialist, and primary care services. Medicare also covers the costs of medications, with patients only required to pay medication dispensing fees once medications have been approved for inclusion on the Pharmaceutical Benefits Scheme [7]. In March 2016, unrestricted access to DAA therapy was introduced through the Australian Pharmaceutical Benefits Scheme [8]. Following the introduction of DAAs, Australia made significant early advancements towards hepatitis C elimination, with over 32,000 people prescribed DAA treatment in 2016 [9]. However, despite granting prescribing authority to a broad range of clinicians, sustained reductions in hepatitis C testing and DAA prescribing means that Australia may miss its elimination targets [10–13]. In 2019, the Australian Government Department of Health released the fifth iteration of the National Hepatitis C Strategy, which provides recommendations for strengthening Australia’s hepatitis C response, including the need to build a strong evidence base to inform the development of models of care in community-based priority settings [14].

Despite comparable treatment outcomes [15, 16], people who inject drugs experience a multitude of system-, provider-, and individual-level barriers to hepatitis C testing and treatment. These include experiences of stigma and discrimination, difficulties accessing and navigating health services which are often disjointed and siloed, and physical and psychological comorbidities [17–19]. While these barriers are multifaceted, a major challenge is that hepatitis C treatment providers are not always focused or aware of the needs of people who inject drugs [20, 21].

The effective provision of hepatitis C care is dependent on sustained engagement across the cascade of care. Hepatitis C models of care often experience substantial loss to follow-up, particularly when they are situated in specialist tertiary settings [22]. Understanding which hepatitis C models are effective in engaging and retaining people at-risk of hepatitis C in testing and treatment is therefore critical to achieving global elimination goals. There is a growing body of evidence demonstrating that establishing nurse-led hepatitis C testing and treatment pathways in community settings is an effective strategy for overcoming patient- and provider-level barriers [23]. Recent evaluations of hepatitis C models of care in Australia have shown that they can be effectively implemented in community-based settings, including homelessness services [24, 25] and mental health and alcohol and other drug (AOD) settings [26–28]. However, there are limited examples of nurse-led hepatitis C models of care which are implemented across large geographic areas in settings with no previously established community-based hepatitis C testing and treatment pathways.

The aim of this study was to describe the establishment and implementation of a nurse-led hepatitis C model of care delivered in the priority settings of AOD services, needle and syringe programmes (NSPs) and mental health services, with a focus on the reach and establishment of the model, and the benefits and challenges of implementation. We further aimed to assess the effectiveness of the model to provide hepatitis C testing and treatment pathways in community-based settings, and to engage people diagnosed with hepatitis C in treatment and retain them in care.

## Description of the project

### Study setting

Tasmania (traditionally named lutruwita) is Australia’s largest island, 64,000 square kilometres in area [29]. At the start of 2020, it was estimated that 2,151 of the estimated 555,975 people living in Tasmania had chronic hepatitis C, corresponding to a prevalence of approximately 0.4% [30, 31]. At the outset of the Tasmanian Eliminate Hepatitis C Australia Outreach (TEHCAO) project, more than half of hepatitis C care in Tasmania occurred in specialist hospital settings, limiting access for people at-risk of hepatitis C who experience challenges accessing the Tasmanian hospital system [32].

### Project design

The TEHCAO project is a nurse-led outreach model of care servicing hepatitis C priority populations and community settings, established in May 2020 through the Sexual Health Service Tasmania (SHST), a government-funded, state-wide health service and funded by the Paul Ramsay Foundation through the Eliminate hepatitis C (EC) Australia partnership. The TEHCAO project was designed to address gaps in community-based DAA prescribing by establishing nurse-led hepatitis C care pathways in priority settings across the state which did not have, or did not prioritise, hepatitis C testing and treatment care pathways. Settings included AOD services, NSPs and mental health services. Services provided by the TEHCAO project included hepatitis C education for staff and clients and clinical care across the hepatitis C cascade of care, including testing, treatment and post-treatment support.

### Human resources

A senior Clinical Nurse Consultant (CNC) was employed to implement the TEHCAO project (0.8 full-time equivalent). In the Australian context, the role of a CNC is a specialised and advanced nursing role that differs from other nurse and health clinician roles in terms of responsibilities, and scope of practice. The role of the CNC often includes coordinating the delivery of care across multiple systems and services, whilst employing a flexible and autonomous approach to practice [33]. In Tasmania, CNC typically have at least five years full-time experience post-registration.

### Identification of eligible services

In the three months prior to the implementation of the TEHCAO project, the CNC conducted a state-wide scoping exercise of primary care services, AOD services, mental health services and NSPs to identify potential partners for the delivery of hepatitis C care. Primary care, NSP and AOD services were selected based on their provision specialised health and harm reduction services for people who inject drugs. Mental health services were selected based on the high prevalence of hepatitis C among people diagnosed with mental illness [34, 35]. For each service, a site assessment was conducted which collected information to inform the initial prioritisation and implementation of the model of care, including an evaluation of existing hepatitis C care pathways, the availability of an onsite clinical space, the geographic location and the caseload of the service. Once the TEHCAO project commenced, further services continued to be identified and recruited throughout the implementation of the project.

### Settings – Nurse-led hepatitis C models

#### Drop-in clinics at NSPs

**Fig. 1:**
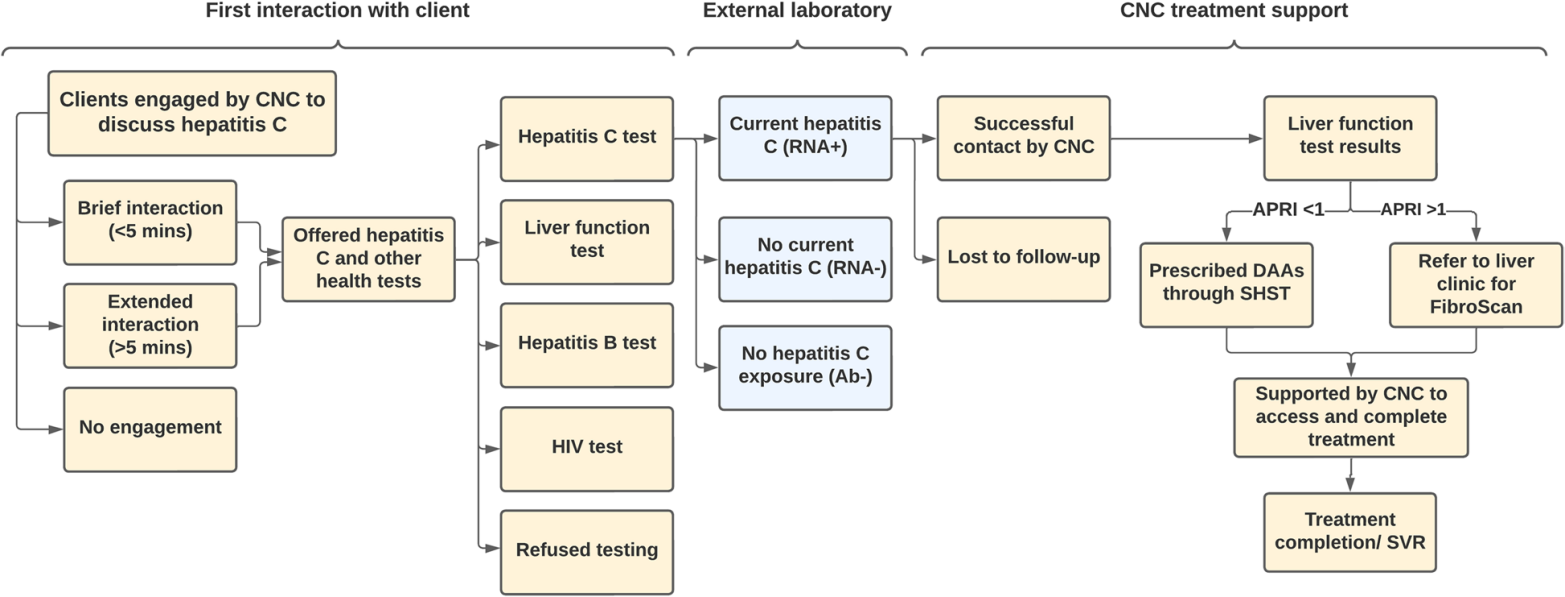
NSP hepatitis C care pathway. NSP: Needle and syringe programme; CNC: Clinical Nurse Consultant; DAA: Direct-acting antiviral; SVR: Sustained virological response; APRI: AST to Platelet Ratio Index. Note: APRI score is a non-invasive test with a score > 1 indicating possible liver fibrosis

Regular drop-in clinics were held by the CNC at participating NSPs, starting in July 2020. Health promotion materials were made available for two weeks before the drop-in clinics commenced, and service staff received education about hepatitis C testing and treatment from the CNC. Clients who were engaged by the CNC were provided with hepatitis C and harm reduction education (referred to onwards as ‘client interactions’), and were offered hepatitis C, liver function and other blood borne virus testing (hepatitis B and HIV). Clients who received hepatitis C testing were offered a $20 AUD gift voucher (funded by the NSP and administered by NSP staff), to reimburse them for their participation. Venipuncture was conducted onsite by the CNC, with all samples sent to an external laboratory for processing. Hepatitis C testing included an initial hepatitis C antibody test, with a hepatitis C RNA test automatically conducted if the initial hepatitis C antibody test was positive (Fig. 1).

#### Mental health and alcohol and other drug services

**Fig. 2:**
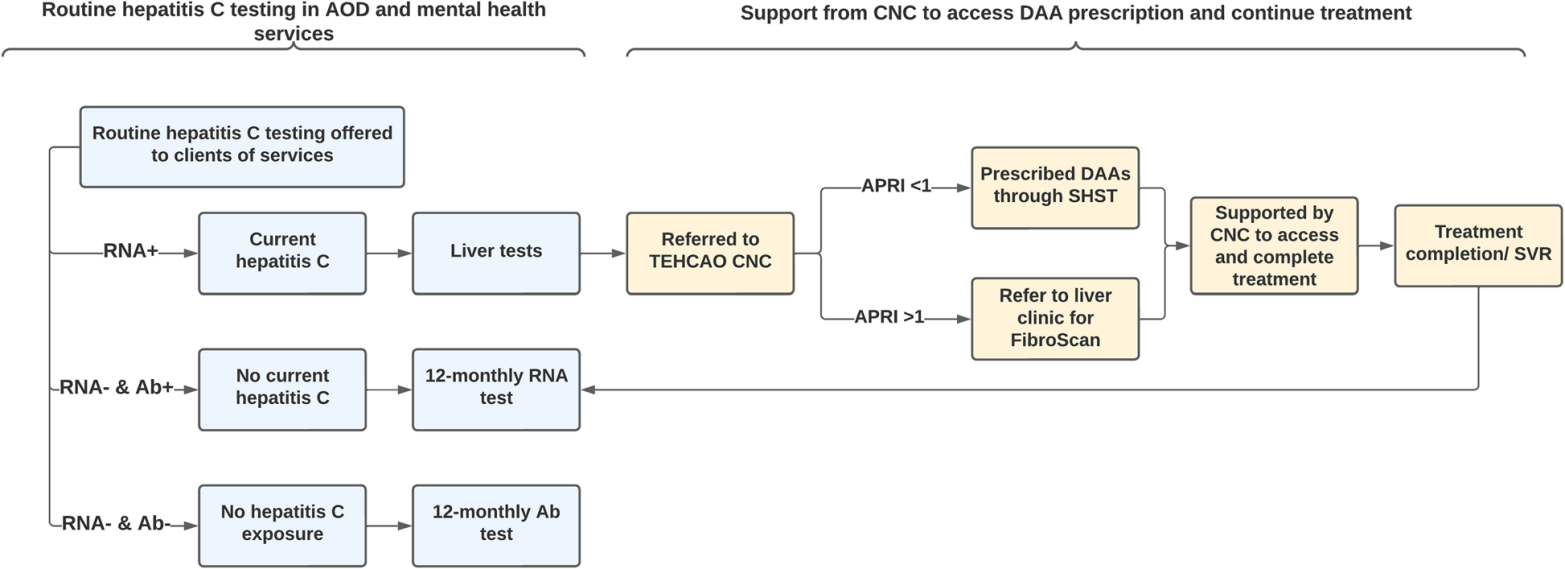
Mental health services and AOD screening and referral pathway. NSP: Needle and syringe programme; CNC: Clinical Nurse Consultant; DAA: Direct-acting antiviral; SVR: Sustained virological response; APRI: AST to Platelet Ratio Index. Note: APRI score is a non-invasive test with a score > 1 indicating possible liver fibrosis

The CNC worked collaboratively with inpatient and community mental health services and AOD services to increase awareness of hepatitis C among staff, and increase testing coverage within services. With support from the CNC, participating AOD and mental health services enhanced their standard of care to include routine, onsite hepatitis C testing for clients (Fig. 2). This included hepatitis C screening for all new clients at admission, and routine annual screening for all clients. To improve the sustainability of the model and develop the hepatitis C workforce in AOD and mental health settings, hepatitis C tests were conducted by service staff, with the CNC providing additional testing capacity when required. Hepatitis C treatment referral pathways between AOD and mental health services and SHST were established, whereby clients who were diagnosed with chronic hepatitis C at participating services were referred to the CNC for hepatitis C treatment support (Fig. 2).

To support services to standardize routine hepatitis C testing, regular education sessions were conducted by the CNC within participating AOD and mental health services. Workforce development activities included tailored support in groups of one to three staff, and group hepatitis C education sessions. Tailored support included working with service staff who were not experienced or lacked confidence performing venipuncture, and supporting them to have conversations with clients about hepatitis C risk factors, testing procedures, and DAA treatment options. Group education sessions were typically in-person and lasted approximately 20 min. Material covered during these sessions included information about risk factors for hepatitis C, testing and treatment options, and Australia’s 2030 elimination targets.

#### Impacts to service delivery during COVID-19

Tasmania experienced low community transmission during the implementation of the TEHCAO project, largely due to the State Government’s border closures. Nonetheless, measures to reduce direct COVID-19 exposure and transmission such as social distancing and restricted gathering sizes were implemented, which had some impacts on the TEHCAO project. First, the start date of nurse-led activities was delayed from May 2020 until July 2020. Second, group meetings were conducted online between April 2020 and August 2020. Third, between March 2020 and April 2020, the North-West region of Tasmania experienced a COVID-19 outbreak. Nurse-led activities in the North and North-West regions of Tasmania were therefore delayed, and did not commence until March 2021.

#### Clinical nurse consultant DAA treatment support across NSPs, mental health and alcohol and other drug services

The CNC facilitated access to DAA treatment by presenting the case to a prescriber at SHST who wrote a script based on a desktop clinical review of the client. The pharmacy dispensing cost of the medication (between $6.60 AUD and $42.50 AUD depending on the patient’s eligibility for concession) was compensated for clients, and clients were able to collect their prescription from a pharmacy convenient to them. The CNC provided a flexible, person-centred approach to support clients during their treatment, including delivering medication to clients who experienced difficulty accessing a pharmacy and/or storing the medication, building partnerships with community mental health case workers and case managers so they could facilitate access to the medication, providing reminders to clients to collect medication, providing regular phone calls to clients to check treatment progress, and performing outreach visits with case managers to clients’ homes.

An APRI score (AST to Platelet Ratio Index) was calculated for clients diagnosed with chronic hepatitis C, with an APRI score greater than one indicating evidence of possible liver fibrosis. Clients whose liver function tests indicated possible liver fibrosis were supported by the CNC to attend a hospital liver clinic for a liver fibrosis assessment and shared care with the CNC.

## Evaluation methods

### Evaluation design

This evaluation used a mixed methods approach. Nurse-led activity data were used to describe the establishment and implementation of a nurse-led model of hepatitis C care implemented in AOD, NSP and mental health services. Clinical outcomes data were used to assess the effectiveness of the model in engaging and retaining clients in hepatitis C treatment. Semi-structured interviews with staff and healthcare providers were used to identify implementation learnings and understand their perspectives and reflections on the model of care. The data used in this study were collected between July 2020 and July 2022.

Nurse-led activities data and clinical outcomes data were analysed using Stata Version 17.0 for Windows [36]. The coding frameworks for semi-structured interview data were created using NVIVO-12 [37].

The findings of this study were reported according to the Standards for Quality Improvement Reporting Excellence (SQUIRE 2.0) guidelines [38].

### Nurse-led activities data

To describe the implementation of the model, analyses included onsite service delivery data and phone call activity data collected by the CNC. Onsite service delivery data included the number of sites engaged, the type of onsite service delivery (clinical tasks, education sessions, both), the number of staff who attended education sessions (1–3 staff, 4–10 staff, 11 + staff), the number of client interactions (< 5 min, 5–20 min) and the number of clients who received a hepatitis C test. Phone call activity data included the total number of phone calls, phone call duration (< 5 min, 5–10 min, > 10 min) and the purpose of the phone call. Nurse-led activity data were entered daily using Research Electronic Data Capture (REDCap) software [39].

### Treatment outcomes and client characteristics

To characterise clients, understand service reach, and evaluate the effectiveness of the model of care to retain clients in hepatitis C treatment, a retrospective chart review of the SHST patient management system was conducted by the CNC for clients who were treated through the TEHCAO project. Treatment outcomes included treatment commencement (collection of first month supply of medication), treatment completion (collection of final month supply of medication) and sustained virological response (SVR) (negative hepatitis C RNA test 12 weeks after completion of treatment). Other data available from the patient management system included age (< 40, ≥ 40), sex (male, female), current prescriptions for opioid agonist therapy (OAT), antipsychotics, antidepressants and mood stabilisers, whether clients reported stable housing (yes, no), AST to Platelet Ratio Index (APRI)score (> 1, ≤ 1) and geographic residential location.

### Healthcare provider interviews

To understand the benefits and challenges experienced during the implementation of the project, healthcare providers and staff from partnered services were invited to participate in a semi-structured interview via telephone or videoconference. Participants were eligible if they were employed at one of the partnered services and had ongoing involvement in the project. Eligible staff were sent participation information and were invited to ask questions about the study. Interviews were scheduled with participants after receipt of written consent.

One author (SC) conducted all the semi-structured interviews [40]. Participants verbally reconfirmed their consent at the start of the interview. Interviews focused on the experiences of participants during the implementation of the TEHCAO project, the challenges and successes of the model of care, how the model could be improved, and the role of nurse-led models of care in achieving hepatitis C elimination in Tasmania. The interview schedule is provided as a supplementary file (Table S1). Interviews were digitally recorded and transcribed verbatim. Transcripts were de-identified and participants could nominate to receive a copy of their transcript for data validation.

An inductive thematic analysis approach [41] was used to analyse the semi-structured interviews. The coding framework was independently produced by the qualitative researcher who conducted the interviews (SC) and was validated by another author (LW) to ensure appropriate and consistent coding.

### Ethics committee approval

This study was approved by the University of Tasmania Research Ethics Review Committee (#26,681). All participants involved in qualitative interviews provided informed consent prior to data collection commencing. Prior to data collection, interview participants were provided with a participant information outlining the aims and structure of the semi-structured interviews. The quantitative component of the evaluation analysed routine Quality Assurance data collected during standard clinical care at SHST. Therefore, no consent process was in place, and a waiver of consent was granted to analyse project data in accordance with Australian guidelines [42]. All methods were performed in accordance with the relevant guidelines and regulations.

## Results

### Nurse-led activities data

A total of 25 services were identified during the initial state-wide scoping exercise which occurred prior to the commencement of the model of care, of which 12 agreed to participate. A further 31 services were identified and participated in the project during its implementation, resulting in a total of 43 participating services.

Between July 2020 and July 2022, there were a total of 219 days of onsite service delivery across the 43 participating services, of which 142 (65%) included clinical care, 45 (21%) included nurse-led hepatitis C education sessions with staff and 32 (13%) included both (Table 1). The highest proportion of days of clinical care occurred in NSP services (93/142, 67%). A total of 77 hepatitis C education sessions were conducted by the CNC, the majority in mental health settings (54/77, 70%). A total of 345 staff attended education sessions. Most nurse-led education sessions had 1–3 attendees (47/77, 61%), and approximately one in ten education sessions were larger groups of more than ten staff (9/77, 12%). There were a total of 695 interactions’with clients, of which 200 (29%) lasted five minutes or less and 495 (71%) lasted more than five minutes. A total of 383 clients received a hepatitis C test from the CNC. The highest number of interactions with clients occurred in NSPs, with 617 (89%) clients engaged by the CNC, and 325 (85%) clients tested for hepatitis C.

**Table 1:** Onsite service delivery, overall and by setting, July 2020 – July 2022

	NSP, n (%)	AOD, n (%)	MHS, n (%)	Other, n (%)^a^	Total, n
Sites engaged	11 (26)	5 (12)	18 (42)	9 (21)	43
Days of service delivery					
Clinical care	93 (67)	11 (8)	2 (1)	32 (23)	142
Nurse-led education	0 (0)	1 (2)	40 (89)	4 (9)	45
Both	11 (34)	7 (22)	14 (44)	0 (0)	32
Total	104 (48)	19 (9)	56 (26)	36 (17)	219
Nurse-led education					
1–3 staff attended	10 (21)	6 (13)	28 (60)	3 (6)	47
4–10 staff attended	1 (5)	2 (10)	18 (86)	0 (0)	21
11 + staff attended	0 (0)	0 (0)	8 (89)	1 (11)	9
Total	11 (14)	8 (10)	54 (70)	4 (5)	77
Total staff attended	16 (5)	27 (8)	271 (79)	31 (9)	345
Client interactions					
< 5 min	198 (99)	2 (1)	0 (0)	0 (0)	200
5 + minutes	419 (85)	24 (5)	31 (6)	21 (4)	495
Total	617 (89)	26 (4)	31 (4)	21 (3)	695
Clients tested by CNC^b^	325 (85)	17 (4)	27 (7)	14 (4)	383

*NSP* needle and syringe programmes, *AOD* alcohol and other drugs, *MHS* mental health service, *CNC* clinical nurse consultant

^a^Other sites include tertiary and specialist services, homelessness support services and sexual health services

^b^Includes hepatitis C antibody and hepatitis C RNA testing

A total of 233 phone calls were made throughout the project, most made to clients (Table S2). The highest proportion of phone calls to clients occurred in NSP services (131/186, 70%), and the highest proportion to service staff and case managers occurred in mental health services (21/47, 45%). Approximately half of all phone calls lasted between five to ten minutes (103/233, 44%), and approximately a third lasted more than ten minutes (66/233, 28%). The most common purpose for phone calls to clients was to provide treatment support and schedule appointments, and the most common purpose for phone calls to staff was to discuss a patient.

### Treatment outcomes and client characteristics

Among the 325 clients who received hepatitis C testing in NSP settings, 30 were identified as hepatitis C RNA positive (Tables 1 and 2). A further 45 clients were identified as hepatitis C RNA positive across the other settings, resulting in a total of 75 clients identified as hepatitis C RNA positive between July 2020 and July 2022. The number of clients who received a hepatitis C test by service staff in non-NSP settings was not recorded.

**Table 2:** Hepatitis C treatment outcomes, overall and by setting, July 2020 – July 2022

Setting	Diagnosed chronic hepatitis C, n	Treatment start, n (%)	Treatment completion, n (%)	SVR, n (%)
NSP	30	28/30 (93)	25/30 (83)	11/30 (37)
AOD	18	17/18 (94)	15/18 (83)	10/18 (56)
MHS	14	14/14 (100)	12/14 (86)	11/14 (79)
Other^a^	13	12/13 (92)	10/13 (77)	7/13 (54)
Total	75	71/75 (95)	62/75 (83)	39/75 (52)

*SVR* sustained viral load, *NSP* needle and syringe programmes, *AOD* alcohol and other drugs, *MHS* mental health service

^a^Other sites include tertiary and specialist services, homelessness support services and sexual health services

Among the 75 clients who tested hepatitis C RNA positive, 95% (71/75) commenced treatment, 83% (62/75) completed treatment, and 52% (39/75) had an SVR test (Table 2). All clients who received an SVR test attained viral clearance. The proportion of clients who started and completed treatment was similar between settings. The highest proportion of clients who were tested for and achieved SVR was among clients diagnosed with hepatitis C in mental health services (11/14, 79%), and the lowest proportion was among clients diagnosed with hepatitis C in NSP services (11/30, 37%).

Among clients treated through the TEHCAO project, two-thirds were over 40 years old (50/75, 67%), and two-thirds were male (52/74, 70%) (Table 3). Clients were commonly prescribed treatments for comorbidities, with 18 (24%) prescribed antipsychotics, 18 (24%) prescribed antidepressants and six (8%) prescribed mood stabilisers. One in three clients were prescribed OAT (24//75, 32%), and one in eight had an APRI score > 1 (10/75, 13%). One in six clients reported unstable housing (12/75, 16%), and two-thirds lived in the Southern region of Tasmania (50/75, 67%). The proportion of clients who started treatment, completed treatment, and received an SVR test was similar across client characteristics, including known predictors of treatment drop-out such as APRI score and unstable housing.

**Table 3:** Characteristics of clients who were hepatitis C RNA positive and treatment outcomes, July 2020 – July 2022^a^

	Diagnosed chronic hepatitis C, n^b^	Treatment start, n (%)^b^	Treatment completion, n (%)^b^	SVR test, n (%)^b^
Total	75	71/75 (95)	62/75 (83)	39/75 (52)
Age group (years)				
< 40	25	24/25 (96)	19/25 (76)	12/25 (48)
≥ 40	50	47/50 (94)	43/50 (86)	27/50 (54)
Sex^c^				
Female	22	21/22 (95)	18/22 (82)	10/22 (45)
Male	52	49/52 (94)	43/52 (83)	28/52 (54)
Prescribed OAT^d^				
No	51	49/51 (96)	43/51 (84)	27/51 (53)
Yes	24	22/24 (92)	19/24 (79)	12/24 (50)
Prescribed antipsychotics^d^				
No	57	53/57 (93)	47/57 (82)	25/57 (44)
Yes	18	18/18 (100)	15/18 (83)	14/18 (78)
Prescribed antidepressants^d^				
No	57	54/57 (95)	45/57 (79)	29/57 (51)
Yes	18	17/18 (94)	17/18 (94)	10/18 (56)
Prescribed mood stabilisers^d^				
No	69	65/69 (94)	57/69 (83)	-
Yes	6	6/6 (100)	5/5 (83)	-
Stable housing^d^				
No	12	12/12 (100)	9/12 (75)	-
Yes	63	59/63 (94)	53/63 (84)	-
APRI score^e^				
> 1	10	8/10 (80)	7/10 (70)	6/10 (60)
≤ 1	65	63/65 (97)	55/65 (85)	33/65 (51)
Region of Tasmania				
Southern	50	50/50 (100)	44/50 (88)	-
Northern	16	15/16 (94)	12/16 (75)	-
North-west and regional/remote	9	6/9 (67)	6/9 (67)	-

*SVR* sustained viral load, *OAT* opioid agonist therapy, *APRI* AST to Platelet Ratio Index

^a^Numbers are suppressed where a cell contains less than five clients to limit individual identification

^b^Denominator for percentages is row total

^c^Sex was missing for one client

^d^At time of hepatitis C RNA positive test

^e^APRI score is a non-invasive test with a score > 1 indicating possible liver fibrosis

## Qualitative outcomes

### Semi-structured interviews

Eleven health professionals were invited to participate in semi-structured interviews, of which ten agreed to participate and were provided with a participation information form. Interviews were conducted between May 2022 and July 2022. Participants involved in semi-structured interviews represented a range of service settings, including community AOD, mental health and harm reduction services, tertiary hospitals, statewide health services, and the state government health department. Participants identified benefits of the model of care when compared to usual practice. Participants also discussed the challenges related to the implementation of the model of care, and to the access and availability of hepatitis C care in Tasmania more generally.

### Benefits and reflections of the TEHCAO project

Participants identified benefits of the TEHCAO project when compared to standard practices of hepatitis C testing and treatment pathways in Tasmania and provided reflections and insights into the experiences of participants involved in implementing the model of care. These benefits and reflections were grouped into three themes: 1) Care is person-centred; 2) Addressing stigma and discrimination; and 3) Organisational impact, success factors and challenges.

#### Theme 1: Care is person-centred

All participants reported that the TEHCAO project allowed the individual circumstances and needs of clients to be identified and addressed and meant that the clients engaged in care in ways that suited them. Features of the model identified as important to the success of the program were, providing a “drop-in” service without appointments and offering longer consultations times when needed. The provision of financial incentives for testing, and covering costs associated with medication dispensing, helped address some clients’ financial barriers to engagement.

“We will pay for the cab fare to attend the clinic to get their bloods taken. At one stage [the nurse] was offering a [supermarket] voucher for people that got tested…I think quite a few of our patients … took advantage of that offer, and had their bloods done.” [Participant 5, AOD setting]

Critically, the CNC was considered a hepatitis C expert among staff at partnering health services and highly skilled at venipuncture, and provided an onsite testing option for clients who previously needed to attend external pathology services. This enabled more clients to be tested and linked to treatment.

“I’ve got a patient that has had hep C for 20 years … I’ve faxed pathology forms to every pathology place … in the hope that she would attend one of those places to have her bloods done, which she hasn’t. But we were able to get [the nurse] here onsite and [she] had the confidence and skillset to be able to take the blood from the patient and be able to link her directly to treatment.” [Participant 6, SHST]

The partnership approach between the CNC and the partnered services improved continuity of care and client linkage to care. Participants reported that the partnership made referral to other services easier, and enabled the development of individualised client support strategies which drew from both local- and state-wide knowledge of health and community services and systems. Because referrals and linkage to care was easier, staff from partnered services became more likely to screen for hepatitis C risks and other health needs because they knew these issues could be followed up onsite with sensitivity and in a timely manner.

“It felt like we were doing a lot more interventions around blood borne viruses in general. … Just having a nurse on site that people felt comfortable with … opened up a lot more conversations. Because people were like, okay, you’re a nurse, you know what you’re talking about medically. I think we have a good rapport with our clients as NSP workers but having that extra layer of a healthcare professional, definitely [helps].” [Participant 10, NSP setting]

“…We’ve been able to link [client name] to treatment [through] the relationships that the … nurse has with the liver clinic …[the nurse] was able to … liaise with all of the important people and pull together a treatment plan really, really quickly. Whereas it was so far outside of our scope that we would have … referred on to the gastro team and then my patient probably wouldn’t have gone…” [Participant 3, AOD setting]

The person-centred nature of the model was perceived by participants to lead to positive outcomes for clients. Participants believed that incentives increased engagement in hepatitis C testing. Services also reported an increase in peer and self-referral for testing as clients became aware of the TEHCAO project.

#### Theme 2: Addressing stigma and discrimination

Most participants perceived that the model of care normalised conversations about hepatitis C and promoted non-judgemental healthcare, primarily through the CNC’s attitude and approach to hepatitis C education and management. Service staff reflected that the CNC provided opportunities for staff to engage in hepatitis C education, helped them to strengthen their organisational practices around hepatitis C and be more confident discussing hepatitis C with clients.

Several participants expressed concern that approaches to hepatitis C education or risk assessment required them to make assumptions about a clients’ lifestyle, which could perpetuate stigma or be perceived by clients to be stigmatising or discriminatory. Participants noted that the implementation of the model of care helped to normalise and standardise hepatitis C care within their service, and therefore the need to make individual assessments about clients.

“…the other barrier is from the clinical point of view, a few of my colleagues are worried that they will come across as being stigmatising if they ask the patients for bloods, especially… the blood borne viruses, so they’re a bit too scared of offending people…” [Participant 5, AOD setting]

Participants reported that for many clients, positive healthcare experiences related to hepatitis C in partnered services improved their confidence to engage with other healthcare services. The ability to see a single healthcare professional for hepatitis C education, testing and treatment reduced the number of healthcare workers that clients had to disclose their hepatitis C status and/or injecting drug use to, which improved client confidentiality and allayed fears of experiencing stigma and discrimination.

“… people are worried about punitive actions … if they're … hepatitis C positive… because of the stigma around … injecting drug use, people are very fearful of giving information … this is between you and the person who's taking your bloods and prescribing the medication. … that's a reassurance within itself....” [Participant 10, NSP setting]

### Theme 3: Organisational impact, success factors and challenges

Most participants reflected that implementing the nurse-led outreach model had additional benefits beyond the delivery of clinical services. The model was viewed as complementary to the health and harm reduction services already available, while also being minimally disruptive to standard organisational operations.

“It’s really just an additional service, so it’s business as usual and it doesn’t disrupt anything … it just works really fluidly.” [Participant 1, NSP setting]

The CNC was also seen as a professional development resource, building staff knowledge and confidence around hepatitis C. This led to staff having more conversations about hepatitis C with clients, and also sharing what they had learnt with each other. Despite their support for the TEHCAO project, participants also identified challenges within the model and system-wide barriers to accessing hepatitis C care.

An important challenge was that only one nurse was employed to deliver the TEHCAO project across Tasmania, leading to a high clinical and administrative workload, and the need to travel across large geographic regions.

“… the fact that she can’t be everywhere at once and … we have seven sites and she has to visit each site … she hasn’t been able to visit all the sites very often, … that’s a staffing issue.” [Participant 1, NSP setting]

Additionally, having different information technology (IT) systems between organisations meant that test results were not always accessible. At times, this led to confusion around whether client information could be shared between the partnered organisations and the CNC.

“… we have a database with patients that have gone missing … and at no stage did we ever actually sit down and go through that database together. So, there wasn't really any allocated time for that, or even clarity about whether that was okay, or whether [the nurse] just needed to start from scratch ...” [Participant 9, Tertiary hospital setting]

A lack of resourcing and capacity to offer hepatitis C testing and treatment pathways in AOD, NSP and mental health settings, which are primarily funded to deliver other services, was identified as a barrier. The CNC provided additional capacity to address this gap in service delivery, particularly when clients required care that was outside the scope of service staff, such as further follow-up and linkage to treatment.

“… the alcohol and drug services …have always been severely underfunded and undermanaged in terms of human resources. So, there's long waiting lists for detox, for example, or for methadone programs” [Participant 9, Tertiary hospital setting]

In Australia, general practitioners (GPs) are able to provide healthcare services at no cost to clients via a government-funded rebate, referred to as “bulk billing” [43]. However, Australia has recently experienced large declines in the number of GPs who offer bulk-billed appointments, particularly in rural and remote areas [44, 45]. In our study, participants highlighted the shortage of bulk billing GPs who were knowledgeable about hepatitis C and confident working with people who inject drugs, as a systemic issue impacting access to hepatitis C care. Although the focus of this project was a nurse-led community-based model, participants recognised the importance of GPs in providing additional options for clients accessing community-based care. However, GP care came with barriers related to access, costs, needing to book appointments, continuity of care, and sometimes poor knowledge about hepatitis C testing and management.

“ … in General Practice people can be given incorrect advice about hepatitis C treatment or the process becomes very difficult. So, they go to one appointment, and then they might get sent [for] bloods, and then they come back, and it wasn’t enough, and they get sent [again], and then they’re … annoyed. Then it might be an out-of-pocket cost and they’re not bulk billed. I think the GP world can be difficult for people to navigate, and the costs can be hidden …” [Participant 6, SHST]

## Discussion

The TEHCAO project successfully implemented a nurse-led hepatitis C model of care in NSPs, AOD services and mental health services across Tasmania, thus establishing a community-based testing and treatment pathway for people living with and at-risk of hepatitis C. The model of care overcame a number of barriers to hepatitis C testing and treatment, and facilitated sustainable improvements in the hepatitis C capabilities of partnered services through enhancing and supporting their hepatitis C and harm reduction activities. There was high retention in treatment among people diagnosed with hepatitis C throughout the project, underscoring the important role nurse-led, person-centred models have in facilitating hepatitis C treatment and care in community settings. A lasting and sustainable outcome of the project was the successful integration of hepatitis C testing pathways into routine clinical care at participating AOD and mental health services. However, given the large number of participating services, and the varied staff and resource capacity of services involved in the project, it was not possible to integrate permanent hepatitis C care pathways into all participating services.

The characteristics of the TEHCAO project which contributed to its success closely align with Australian and international recommendations for the delivery of hepatitis C care within primary care and community-based settings [1, 46–49]. First, the TEHCAO project provided hepatitis C care in community-based health and harm reduction services which were familiar and accessible to people who inject drugs. Second the collaborative, partnership approach that was employed by the project led to the establishment and improvement of hepatitis C care pathways within participating services. Third, the TEHCAO project used a person-centred approach which recognised the varied needs of clients, including repeat or delayed testing and treatment for clients when required, storing and delivering medication on behalf of clients and partnerships with community mental health case workers and case managers to ensure the needs of clients were met. These findings underscore the importance of recognising the role of nurse-led, community-based, hepatitis C models of care and including them in in contemporary hepatitis C guidelines.

Resourcing and logistical constraints mean that nurse-led hepatitis C models of care often operate within a single, or a limited number of services situated in urban, inner-city areas. Careful planning through detailed site assessments, collaborative partnerships with services and flexible testing and treatment pathways enabled the TEHCAO project to implement a model of care across a large geographic area and a variety of health services and settings. Importantly, implementing the project in both urban and non-urban areas did not negatively impact treatment outcomes, and the proportion of clients diagnosed with chronic hepatitis C who were retained in treatment was comparable to other nurse-led models of care, both in Australia [25–27, 50, 51] and globally [23, 52–54]. Our evaluation demonstrates that nurse-led hepatitis C models of care can overcome patient, provider and system related barriers, including geographic barriers.

Whilst the TEHCAO project successfully established hepatitis C testing and treatment pathways in community settings and was effective in engaging and retaining people in care, our evaluation identified several risks and challenges involved in its implementation. First, the large geographic area within which the project was implemented required the CNC to travel significant distances visit partnered services, which increased the workload. Second, there were challenges locating GPs who were willing to provide hepatitis C care for people who inject drugs, particularly in rural and remote areas. Whilst this barrier was addressed through the establishment of referral pathways with SHST, it meant that many clients had no localised hepatitis C care pathways and were dependent on the CNC for treatment support. Third, the project was highly dependent on the employment of a highly skilled CNC, who had previously worked with people who inject drugs, and had experience supporting people with hepatitis C treatment in general practice, custodial and mental health settings. Shortcomings in the knowledge and confidence of clinicians to provide hepatitis C care in community settings were reported by interview participants of this study, and have also been identified in previous Australian studies [55, 56]. Consequently, the project was highly dependent on the continued employment and availability of the CNC, representing a significant risk to the successful implementation of the project.

Whilst Australia has experienced sustained reductions in hepatitis C prevalence and incidence following the widespread availability of DAAs, there are a number of persisting challenges related to the diagnosis and treatment of the remaining people living with hepatitis C [17]. For many people still living with hepatitis C in Australia, competing health and social priorities due to previous experiences of stigma and discrimination, social disadvantage and substance dependence means that many people remain doubtful, uncertain, and unaware of hepatitis C treatment [57]. This is evident within the TEHCAO project, with many of the clients who were supported through treatment experiencing mental health problems and housing instability. Consequently, the time, energy and compassion required to engage and retain people living with hepatitis C in treatment is considerable. However, despite these challenges, the TEHCAO project successfully supported clients to access and complete hepatitis C treatment by offering flexible service delivery and establishing strong partnerships with community-based services. These findings provide important insights for countries moving towards elimination and seeking to devolve hepatitis C care from the hospital to the primary and community care system.

## Recommendations for practice

As countries continue to strive towards hepatitis C elimination, the financial and political investment required to find and treat the remaining people living with hepatitis C will continue to increase [57, 58]. It is therefore important that effective, evidence-based models of care which adhere to contemporary hepatitis C testing and treatment guidelines receive sustained investment and support. Funding long-term roles for nurses in community-based settings is critical, as is recruiting nursing staff who are qualified to deliver hepatitis C care across the cascade of care, and are capable of liaising between community and tertiary settings to facilitate the management of patients with complex social and medical needs. Further, strengthening the hepatitis C workforce and developing the capacity of non-specialist healthcare professionals working in priority settings such as AOD services, NSPs and other harm reduction services and primary care remains a priority. Finally, future hepatitis C models of care should adopt a partnership approach during their design and implementation, which includes building collaborative relationships with services, and identifying staff within services with the enthusiasm and clinical skills to implement hepatitis C care. While this project has demonstrated the importance of a partnership approach, ongoing challenges related to staffing and other resourcing underscores the need for administrative and project management support when coordinating outreach models of care delivered across a large number of services and across a large geographic area.

## Limitations

To our knowledge, this is the first evaluation of a nurse-led model of hepatitis C care implemented across Tasmania. The participants interviewed represented a diverse range of organisations and were able to provide experience and knowledge around delivering hepatitis C services across community, hospital, and health policy levels. Although participants interviewed represented a diverse range of services, a key limitation of the evaluation is that clients of partnered services were not interviewed. This meant that findings of the evaluation were reliant on the experiences and perspectives of staff involved in the implementation of the model and delivery of services, and did not include the experiences or perspectives of people who inject drugs or people living with hepatitis C. We recommend that experiences of people accessing services are included in the evaluation of future, similar models of care.

Second, $20 AUD gift vouchers and script payments were provided by a state-wide NSP service to clients as an incentive to participate in hepatitis C testing and treatment. As these financial incentives were not provided by the TEHCAO project, we were unable to measure the total number of incentives that were provided, or the extent to which these incentives may have influenced the success of the project.

Third, Burnet staff provided project management support and were responsible for the evaluation of the project. The Burnet Institute is an independent, not-for-profit research and global health institute which supports healthcare interventions in Australia and globally. In 2019, the Burnet Institute received fundings from the Paul Ramsay foundation to implement a national Australian hepatitis C campaign, which included funding workforce development projects in geographic areas with less capacity and greater need. While Burnet staff members were able to provide a wealth of support to the project team, including cross-sector and research knowledge, it is important to acknowledge that this involvement may have led to more favourable reporting of evaluation outcomes, or less willingness for interview participants to report negative experiences. This limitation has been managed by keeping Burnet staff responsible for the collection and analysis of evaluation data separate from project management activities, and bringing in staff with no pre-existing relationships with participants to conduct interviews and analyse the qualitative data (authors SC and LW).

## Conclusion

The results of this study indicate that nurse-led hepatitis C models of care can feasibly and effectively establish hepatitis C testing and treatment pathways within a large geographic area and across multiple priority settings. However, ongoing challenges related to resourcing, staffing, and working across a large number of siloed services remain, thus reducing the sustainability of the model. These findings reinforce the need for governments and health authorities to provide long-term funding for similar outreach models of care in community settings if they wish to achieve hepatitis C elimination, both in Australia and globally.

## Availability of data and materials

The datasets analysed during the current study are not publicly available as per University of Tasmania Research Ethics Review Committee approval. Analysis code is available upon reasonable request to the corresponding author.

### Supplementary Information

**Additional file 1.**

## Data Availability

The datasets analysed during the current study are not publicly available as per University of Tasmania Research Ethics Review Committee approval. Analysis code is available upon reasonable request to the corresponding author.

## Abbreviations

AOD: Alcohol and other drug
CNC: Clinical Nurse Consultant
DAAs: Direct Acting Antivirals
GPs: General practitioners
RNA: Ribonucleic acid
NSPs: Needle and syringe programs
SHST: Sexual Health Service Tasmania
SVR: Sustained virologic response
TEHCAO: Tasmanian Eliminate Hepatitis C Australia Outreach project
