# Supplementary material for: Evaluation of a person-centred, nurse-led model of care delivering hepatitis C testing and treatment in priority settings: a mixed-methods evaluation of the Tasmanian Eliminate Hepatitis C Australia Outreach Project, 2020–2022
Source: BMC Public Health. 2023 Nov 20;23:2289. doi: 10.1186/s12889-023-17066-9 (PMC10662700; doi:10.1186/s12889-023-17066-9)
Supplement: Supplementary file 1 — Additional file 1. [file 12889_2023_17066_MOESM1_ESM.docx]

**S1.** Semi-structured interview schedule

**Work as Project Staff**

- To help us understand your experiences and perspectives, please describe your role and experience at your service
  - *Prompt: Position, tasks & responsibilities, time working in role and with service*
- What do you think are some of the barriers and enablers for people being tested or treated for hepatitis C in Tasmania?

**Reflections on hepatitis C outreach model of care model**

- Why did you feel that there was a need for a hepatitis C outreach model of care at your service?
- What were the main successes of the outreach model?
  - *Prompt: Were there changes in the numbers of people who received hepatitis C care?*
  - *Prompt: What were the facilitators?*
- What were some of the challenges of the outreach model?
- What would you change to the outreach model? Describe.
- *Prompt: What ways do you believe the outreach model could be improved?*
- Do you think outreach models of care should be routinely offered in services/settings similar to yours?
  - *Prompt: Why/why not?*

**Learnings**

- Can you comment on any broader social impacts that the hepatitis C outreach model has had for the people accessing your service?
- Did the hepatitis C outreach model overcome any barriers to accessing hepatitis C care in Tasmania?
  - *If yes: What were these barriers and how did the model overcome them?*
- Have you learnt or gained anything professionally from the outreach model that was implemented in your service?
  - *If yes: What have you learnt or gained?*
- Is there any advice or lessons that you have learned which you think would help other organisations seeking to establish outreach models of care at their service?
- What role do you see outreach models of care having in HCV elimination efforts?
  - *Prompt: How could this be supported/implemented?*

**Final question**

- Is there anything that we haven’t covered that you would like to add?

**S2.** Duration, recipient and purpose of phone calls, overall and by setting, July 2020 – July 2022

|  | NSP,  n (%) | AOD,  n (%) | MHS,  n (%) | Other,  n (%)^1^ | Total, n |
| --- | --- | --- | --- | --- | --- |
| Total phone calls | 144 (62) | 12 (5) | 54 (23) | 23 (10) | 233 |
| Phone call recipient |  |  |  |  |  |
| Client | 131 (70) | 7 (4) | 33 (18) | 15 (8) | 186 |
| Staff member/case manager | 13 (28) | 5 (11) | 21 (45) | 8 (17) | 47 |
| Phone call duration |  |  |  |  |  |
| <5 minutes | 38 (59) | 7 (11) | 15 (23) | 4 (6) | 64 |
| 5–10 minutes | 64 (62) | 2 (2) | 26 (25) | 11 (11) | 103 |
| >10 minutes | 42 (64) | 3 (5) | 13 (20) | 8 (12) | 66 |
| Purpose of phone calls to clients^2^ |  |  |  |  |  |
| Hepatitis C education | 6 (38) | 2 (13) | 2 (13) | 2 (13) | 16 |
| Inform of test results | 58 (97) | 0 (0) | 1 (2) | 1 (2) | 60 |
| Treatment support | 72 (62) | 7 (6) | 28 (24) | 9 (8) | 116 |
| Schedule appointment | 75 (70) | 2 (2) | 18 (17) | 12 (11) | 107 |
| Purpose of phone calls to staff^2^ |  |  |  |  |  |
| Hepatitis C education | 3 (27) | 0 (0) | 4 (36) | 4 (36) | 11 |
| Client referred to project CNC | 3 (43) | 1 (14) | 3 (43) | 0 (0) | 7 |
| Discuss patient | 13 (30) | 5 (11) | 19 (43) | 7 (16) | 44 |
| NSP: needle and syringe programmes; AOD: alcohol and other drugs; MHS: mental health service; CNC: clinical nurse consultant  ^1^ Other sites include tertiary and specialist services, homelessness support services and sexual health services  ^2^ Purpose of call can be in multiple categories | | | | | |
